# Glycerine

**Published:** 1867-12

**Authors:** 


GLYCERINE,

As an article of food, as a nutrient, is well worthy of being brought into public notice. Sweet oil in Palestine and other old countries, has for ages been used as an article of daily food, and glycerine may be considered as the essence of pure "sweet", that is "olive oil," they being one and the same; it is a perfectly neutral and bland fluid, and the most penetrating perhaps in all nature. Oil itself will permeate where water will not; and glycerine, which may be considered the ethereal part of oil, has this property to a most remarkable degree; it penetrates the solid bone; if poured into a mixture of blood and matter, such as is expectorated from consumptive lungs, it will get in between the globules of each and show them with great distinctness; being thus penetrating, it is the very best application for all feverish sores, for inflamed or dry surfaces simply from its quality of penetration and want of evaporatability; the first and highest value of any poultice is its capability of keeping moist for the longest time; no one ever thinks of a dry poultice; glycerine keeps a part moist longer than any substance known, hence its value as above, mixed with an innoxious dry powder, called sub-nitrate of Bismuth, so as to make a thin paste or poultice. It is one of the very best applications known for

